# Floral scent changes in response to pollen removal are rare in buzz-pollinated *Solanum*

**DOI:** 10.1007/s00425-024-04403-4

**Published:** 2024-06-03

**Authors:** C. Douglas Moore, Dudley I. Farman, Tiina Särkinen, Philip C. Stevenson, Mario Vallejo-Marín

**Affiliations:** 1 Biological and Environmental Sciences, University of Stirling, Stirling, FK9 4LA UK; 2 Natural Resources Institute, University of Greenwich, Kent, ME4 4TB UK; 3 Royal Botanic Garden Edinburgh, 20A Inverleith Row, Edinburgh, EH3 5LR UK; 4 Royal Botanic Gardens, Kew Green, Kew, Richmond, Surrey TW9 3AE UK; 5 Present Address: Department of Ecology and Genetics, Evolutionary Biology Centre, Uppsala University, 752 36 Uppsala, Sweden

**Keywords:** Chemical ecology, Concealed reward, Floral scent, Linalool, Plant–pollinator signalling, Plant ecology, Pollination, Poricidal flower, Volatile organic compound

## Abstract

**Main conclusion:**

One of seven *Solanum* taxa studied displayed associations between pollen presence and floral scent composition and volume, suggesting buzz-pollinated plants rarely use scent as an honest cue for foraging pollinators.

**Abstract:**

Floral scent influences the recruitment, learning, and behaviour of floral visitors. Variation in floral scent can provide information on the amount of reward available or whether a flower has been visited recently and may be particularly important in species with visually concealed rewards. In many buzz-pollinated flowers, tubular anthers opening via small apical pores (poricidal anthers) visually conceal pollen and appear similar regardless of pollen quantity within the anther. We investigated whether pollen removal changes floral scent composition and emission rate in seven taxa of buzz-pollinated *Solanum* (Solanaceae). We found that pollen removal reduced both the overall emission of floral scent and the emission of specific compounds (linalool and farnesol) in *S. lumholtzianum*. Our findings suggest that in six out of seven buzz-pollinated taxa studied here, floral scent could not be used as a signal by visitors as it does not contain information on pollen availability.

**Supplementary Information:**

The online version contains supplementary material available at 10.1007/s00425-024-04403-4.

## Introduction

Scent is a key floral trait that can influence plant reproductive success by attracting pollinators or manipulating their behaviour and learning (Wright and Schiestl 2009; Russell et al. 2018). Floral scents comprise a mixture of volatile organic compounds (VOC), of which > 2000 have been identified, and may have broad or specific activity (Knudsen et al. 2006; El-Sayed 2019; Gfrerer et al. 2021). Pollinators can detect the presence and determine the relative abundance of VOC, allowing them to discriminate between complex scent blends when making foraging decisions (Byers et al. 2014; Zhao et al. 2022). Therefore, the diversity and abundance of VOC allows flowers to attract specific pollinators and elicit specialized behaviours, such as copulation in sexual mimicry (Schiestl et al. 2003), or vibrations in buzz-pollination (Russell et al. 2018).

Recent advances in floral chemical ecology demonstrate that scent is a dynamic trait that varies across space and time, allowing plants to modulate where and when signals are emitted (Burdon et al. 2015, 2020; García et al. 2021). Spatio-temporal analyses of floral VOC have revealed functions beyond pollination, such as defence against predators (Scala et al. 2013), parasites (Morinaga et al. 2009), and pathogens (Huang et al. 2012). Temporal variation in floral scent is found in many pollination systems and includes daily cycles, where peak emission of VOC coincides with key pollinator foraging activity (Raguso and Willis 2005; Morinaga et al. 2009). Longer term changes include community scale effects caused by drought (Jaworski et al. 2022) or where plants flowering early in the season, when pollinators are rarer, produce stronger scents than those flowering when pollinators are in abundance (Filella et al. 2013). Permanent floral scent changes occur in response to herbivory (Kessler et al. 2011) and florivory (Vega-Polanco et al. 2020), as well as post-pollination to protect seeds from predation (Theis and Raguso 2005; Burdon et al. 2015), thus impacting reproductive success. Experimental studies have also shown that floral scent attracts pollinators across spatial scales: airborne scent plumes act at long range (Dobson 1994), scent guides on petals orient bees at medium range (Dötterl and Jürgens 2005), and scented rewards act at close range (e.g. nectar or pollen; Dobson et al. 1999; Burdon et al. 2020).

Variation in floral scent composition may occur based on floral reward availability and may lead to compound concentrations rising or falling as rewards are depleted and replenished (Wright and Schiestl 2009; Burdon et al. 2015, 2020). We would expect pollinators to prefer scents associated with rewards, because this facilitates more efficient foraging by avoiding depleted flowers. In fact, fossil evidence suggests that olfactory and visual signals associated with rewards were likely present in the most primitive flower–pollinator interactions (Crepet 1983). That flowers typically produce signals from non-rewarding tissues suggests that scented reward production is either not beneficial or unfeasible (Raguso 2004; Leonard et al. 2011). This may be due to relatively small quantities of reward being unable to emit sufficiently large signals to attract pollinators or that adding secondary metabolites to rewards may render them distasteful or toxic (Stevenson et al. 2017; Jacquemart et al. 2019). Alternatively, rewards may be contained within floral structures for protection (e.g. from environmental damage or detrimental floral visitors), which restrict or prevent signal transmission in one or more senses, e.g. nectar of bilabiate flowers, pollen in poricidal flowers, or both in keel flowers (Buchmann et al. 1983; Westerkamp 1997; Westerkamp and Claßen-Bockhoff 2007).

Poricidal flowers occur in approximately 22,000 species across at least 65 families, including some crops (e.g. tomato and kiwi). In contrast with typically longitudinally dehiscent flowers, poricidal flowers release pollen through pores or slits in response to vigorous vibrations: buzz-pollination (De Luca and Vallejo-Marín 2013). This adaptation effectively restricts pollen access to approximately 74 bee genera, containing ca. 58% of bee species, reported to be capable of buzz-pollination (Cardinal et al. 2018). The enclosing structures of poricidal flowers conceal pollen and always appear full, yet bees visit rewarding flowers preferentially where scent stimulates buzzing (Buchmann and Cane 1989; Russell et al. 2017). As floral scent can change quickly (Farré-Armengol et al. 2014) and bees may use this trait to discriminate between rewarding and unrewarding flowers, avoiding inconsistent or unrewarding flowers (Austin et al. 2019; van der Kooi et al. 2023), we hypothesise that the scent of poricidal flowers varies with pollen availability.

Using *Solanum*, a large genus of nectarless flowers that conceal their pollen inside poricidally dehiscent anthers, we asked whether floral scent changes depending upon reward availability. Floral scent would be expected to fluctuate with the presence of reward in the buzz-pollinated flowers of *Solanum* to provide information on reward availability which bees could use when foraging. We also assessed whether the same scent compounds fluctuate in relation to reward availability across the taxa studied. Analysis of floral scent in seven closely related *Solanum* taxa revealed specific changes in the emission and composition of VOC associated with pollen presence in only one taxon and suggest that floral scent acts as a signal of reward availability in plants with concealed floral rewards only rarely.

## Materials and methods

### Study system

**Fig. 1:**
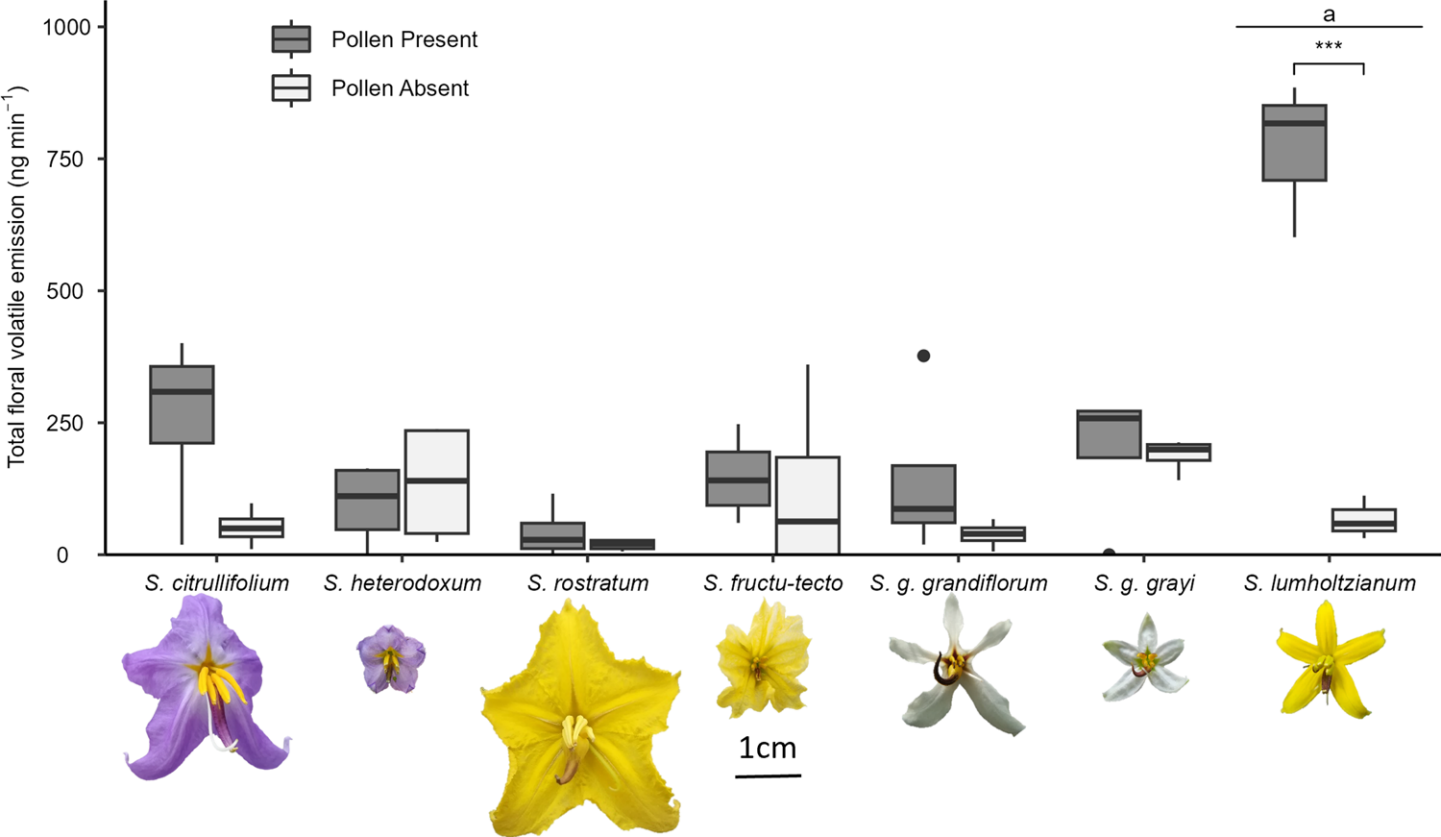
Boxplot of volatile organic compound (VOC) emission rate of *Solanum* section *Androceras* flowers with and without pollen. Mean VOC emission of *Solanum lumholtzianum* flowers was significantly greater than that of all other taxa. The VOC emission rate of flowers containing pollen was significantly higher than in those without pollen in *S. lumholtzianum*. Asterisks signify within-species significant differences (*P < 0.05, ***P < 0.001) and letters denote between-species differences (*P* < 0.05). Sample size: *n* = 4 per treatment per species, except *S.* *lumholtzianum* where *n* = 3

*Solanum* is a large genus (c. 1350 species) used as a model system for investigating buzz-pollination (Särkinen et al. 2013; Russell et al. 2016). *Solanum* flowers are nectarless, often hermaphrodites (Knapp 2002), releasing pollen from small distal anther pores via vibrating insects (King and Buchmann 1996). Here, we studied *Solanum* section *Androceras,* a small clade of self-compatible, annual or perennial herbs native to Mexico and the USA (Stern et al. 2010). We selected seven taxa for analyses of floral scent composition, which have been previously used in detailed analyses of floral variation and mating system (Vallejo-Marín et al. 2014; Kemp and Vallejo-Marín 2021), and the response of floral scent to florivory (Vega-Polanco et al. 2020). *Solanum* *rostratum* (Dunal) and *S. citrullifolium* (A. Braun) produce large floral displays consisting of many flowers open simultaneously, with strong heteranthery present in each flower where one anther is clearly larger than others (Fig. 1) (Stern et al. 2010; Vallejo-Marín et al. 2014). *Solanum fructu-tecto* (Cav.) and *S. heterodoxum* (Dunal) produce small displays consisting of few small flowers open simultaneously across the plant, rarely more than one flower open per inflorescence at any time with shorter anthers and less pronounced heteranthery (Fig. 1). The display size of *S. grayi* is dependent on the presence of the large-displaying *S. lumholtzianum* (Bartlett): in allopatric regions, *S. grayi* produces large displays (*S. grayi* subspecies *grandiflorum*), but in sympatric locations, *S. grayi* produces small displays only (*S. grayi* ssp. *grayi*).

### Plant material

Experimental plants were grown at the University of Stirling. Seeds were collected from wild populations in Mexico between 2007 and 2010, except *S. citrullifolium* which was obtained from Radboud University’s Experimental Garden and GeneBank Solanaceae collection, Nijmegen, The Netherlands (Table 1) (Vallejo-Marín et al. 2014). Germination was stimulated by soaking seeds for 24 h at room temperature in 1000 ppm aqueous gibberellic acid solution (GA3; Sigma-Aldrich, Dorset, UK) in February 2019. Seeds were then drained and planted individually in seed trays (2 × 2 × 5cm) containing Modular Seed Growing Medium Compost (William Sinclair Horticulture PLC, Lincoln, UK) and placed in a growth chamber with 16–24 °C temperature cycle and 75% relative humidity. Plants were illuminated at 4.8–9.5 klx in an 18:6 h light:dark cycle. After 2–4 weeks, s.0eedlings were transferred to individual 1.5 L pots (15 cm diameter) containing a 4:1 mix of All Purpose Growing Medium and Perlite Standard (William Sinclair Horticulture PLC) in a pollinator proof glasshouse. Plants were fertilised weekly with Tomorite Concentrated Tomato Food (Tomorite, Levington, Surrey, UK), watered as needed 3 times a week, maintained between 16 and 25 °C and illuminated for ≥ 16 h per day by sunlight supplemented with compact-fluorescent lights. Flowering started when plants were approximately 75 cm tall and was sustained by removing any seed pods, arising from selfing, with sharp scissors every Friday, at least 72 h before headspace was sampled. Anthesis in Solanaceae often lasts multiple days (Silva-Neto et al. 2016) and lasted for approximately 5 days in the taxa studied if flowers remain unfertilised and plants unstressed (personal observation).

**Table 1:** Seed source locations for *Solanum* section *Androceras* studied

Taxon	Accession	Location	Latitude (N)	Longitude (E)	Elevation (m)	Herbarium specimen
*S. citrullifolium*	199-7-1 199-7-7 199-7-9	Nijmegen, Solanaceae Collection	NA	NA	NA	NIJ: 894750197
*S. heterodoxum*	11-PTEM-14 11-PTEM-15	Teotihuacan Archaeological Site, Mexico State	19.685 19.685	– 98.843 – 98.843	2284 2284	RBGE: E01009043; E01009044; E01009045
*S. rostratum*	10-s-77 10-s-79 10-s-81	San Miguel, Queretaro State	20.901	– 100.705	2033	RBGE: E01009047; E01009048
*S. fructu-tecto*	10-AH-9 11-CU-4	Atitalaquia, Hidalgo State Ciudad University, Mexico City	20.066 19.394	– 99.216 – 99.192	2090 2311	ECOTAP: HET1905
*S. grayi ssp. grayi*	07-s-19-4b 07-s-19-5b 07-s-19-6b	Guamúchil, Sinaloa State	25.340	– 107.951	93	IBUNAM: MEXU1229199
*S. grayi ssp. grandiflorum*	08-s-78 08-s-79	Tejupilco, Mexico State	18.852	100.131	1375	RBGE: E01009041; E01009042
*S. lumholtzianum*	07-s-57 07-s-59	Guamúchil, Sinaloa State	25.340	– 107.951	93	NA

### Floral headspace sampling

Floral headspace samples were collected between May and September 2019. Headspace was continuously sampled from all flowers per plant for 6 h to optimise VOC capture as pilot work by the University of Greenwich indicated low VOC levels were collected over shorter periods from some taxa. Sampling began between 06:00 and 08:00 to cover the period of peak pollinator visitation to wild plants (Solís-Montero et al. 2015) which we hypothesised to coincide with peak attractant VOC emissions (following Muhlemann et al. 2014). Samples were collected by enclosing all flowering inflorescences from a single plant within an odourless 3.2 L PET bag (Multi-Purpose Cooking Bag Sainsbury’s Supermarkets Ltd., London, UK; see Stewart-Jones and Poppy 2006). As flowers in this clade grow on indeterminate inflorescences, stems and unopen flower buds were also enclosed, but leaves were excluded. A mains-operated vacuum-pump (FB65540, Fisher Scientific, Hampton, NH, USA) pulled air through a charcoal filter into the bag to remove contaminants and extracted air through a collection filter at a rate of 2,000 cm^3^ min^−1^. To maintain environmental consistency and so reduce the chance of plant heat stress, samples were collected in a shaded area of the glasshouse plants were grown in. Combined with constant airflow, this precaution prevented condensation forming inside sampling bags, which could interfere with floral scent sampling. Wilting of plant tissue and formation of condensation was never observed. Filters consisted of a 4mm i.d. Pasteur pipette containing Porapak Q (200 mg, 50–80 mesh; Waters, Milford, MA, USA) held between plugs of silanized glass wool. All filters were handled and stored together. On the day of VOC collection, control filters were randomly selected and placed on a sheet of aluminium foil beside the experimental setup for the duration of headspace sampling, but no air was drawn through them. After sampling was complete, all filters were wrapped in clean aluminium foil and transported to the Natural Resources Institute, Chatham Maritime, UK, for analysis.

### Floral headspace sampling experimental treatments

Headspace samples were collected from unmanipulated virgin flowers, and flowers from which pollen was removed. Both treatments were always sampled concurrently from different plants of the same taxon and accession. On the day of headspace collection, pairs of plants with abundant flowers were selected and randomly allocated to either treatment. Pollen was mechanically removed from all flowers of the appropriate plant by vibrating all of a flower’s anthers within a 2ml Eppendorf tube using an electric toothbrush (Braun Oral-B Type 3756; Oral-B, Redwood City, CA, USA) operating at approximately 1000 Hz for 30 s or until pollen expulsion ceased, whichever took longer. Care was taken to ensure that stigmas were excluded from the Eppendorf to avoid pollination which can cause floral VOC changes (Theis and Raguso 2005). Headspace was sampled continuously for 6 h, starting no more than 20 min after pollen removal. Both treatments were simultaneously sampled from separate plants of the same accession. Four samples were collected per treatment per species, except *S. lumholtzianum,* which was only sampled three times per treatment due to difficulty getting sufficient plants to flower. Individual plants were used only once.

### Pollen scent sampling

We hypothesised that pollen was a source of floral VOC, so pollen of *S. citrullifolium*, *S. rostratum*, and *S. lumholtzianum* was extracted as outlined above. Pollen was extracted from all open flowers on a plant, but individual plants were not used elsewhere in this study. VOC was not assessed from flowers of other taxa as a useable amount of pollen could not be collected, because plants produced either too few flowers or flowers contained too little pollen, or both. To ensure sufficient pollen was collected for VOC extraction, pollen from 40 individual flowers was collected and was pooled by species. As very small quantities of pollen were collected (*S. citrullifolium* = 227 mg, *S. rostratum* = 306 mg, and *S. lumholtzianum* = 173 mg), it was decided that headspace sampling may not collect sufficient quantities of volatiles for analysis. Therefore, solvent extraction was used as it likely extracts more volatiles (Kessler and Halitschke 2009) ensuring that we were able to distinguish the volatile chemistry of the pollen from the floral scent and quantify it. After collection, pollen was stored in 2 ml Eppendorf tubes at − 18 °C until scent sampling. To extract pollen VOC, pollen was mixed with 1 ml hexane, left for 1 h, and then centrifuged for 1 min at 2000 *g*. The supernatant was pipetted into clean 2.5 ml glass vials and transferred to the Natural Resources Institute for analysis. No VOC were detected in the solvent extracts from pollen of *S. citrullifolium*, *S. rostratum*, and *S. lumholtzianum*.

### Volatile analysis

Collection filters were extracted in 1ml of dichloromethane. Decyl acetate (5µg) was added to each sample as an internal calibration standard. VOC samples were analysed with flame ionization detection (FID) using an Agilent 7890 GC (Agilent Technologies Inc., Santa Clara, CA, USA), with a split/splitless inlet using helium as the carrier gas (2.4ml min^−1^ flow rate). The column (Agilent DBWax 30 m × 0.32 mm × 250 µm) was held at 60 °C for 2 min before ramping to 240 °C by 6 °C min^−1^. Data were captured and analysed with EZChrom Elite (Agilent). Results were calibrated against pure synthetic standards of the compounds. Peak identities were further confirmed by analysing samples by gas chromatography/mass spectrometry, using a Varian GC (Varian Medical Systems, Palo Alto, CA, USA) coupled to a Varian 2000 ion trap and a DBWax column (Agilent, 30 m × 0.25 mm × 250 µm).

### Analysis

To account for variation in the number of flowers sampled and collection duration (Supplementary Information S1), we standardised data by calculating volatile emission per minute per flower (VOC emission ng^−1^ min^−1^) and used this in all analyses. To determine variation in total floral VOC emission rate between plant taxa and in response to pollen presence, we analysed the per flower total VOC emissions (ng min^−1^) with two-way ANOVA (*aov* function, stats package) using emission as the dependent variable with plant taxa and pollen presence as the explanatory variables (emission ~ taxa × pollen presence). Type III corrections were applied (*Anova* function, car package) to account for an unbalanced design, as one fewer VOC collection was made for *S. lumholtzianum* in each treatment than all other species. Between-species comparisons were explored with Tukey’s honestly significant difference test (Tukey’s HSD: *TukeyHSD* function, stats package). The effect of pollen presence on total VOC emission within species was analysed using Student t tests as these are logical, a priori pairs. To assess whether floral scent composition differed within taxon in response to pollen presence and if floral scent differed between taxa, we used a two-way MANOVA (*manova* function, stats package), analysing emission of the 5 detected VOC per flower (ng min^−1^) as dependant variables with pollen presence and plant taxa as explanatory variables. We further investigated the MANOVA results with two-way ANOVA with Bonferroni corrections (*summary.aov* function, stats package, Alpha = 0.008). R version 4.2.1 (R Core Team 2022) was used for all analyses and figures were generated in R ggplot2 package (Wickham 2016). Assumptions of homogeneity of variance and normally distributed residuals were met for ANOVA and t-test analyses (respectively, Levene’s and Shapiro–Wilk’s tests *P* > 0.05; leveneTest and shapiro.test, *stats* package) and the multivariate equivalent assumptions were also met for MANOVA (assumptions_manova, *micompr* package).

Linalool has two naturally occurring stereoisomers with different biological properties (Raguso and Pichersky 1999), while farnesol has four geometric isomers (Yu et al. 2005). Unfortunately, due to methodological limitations, we were unable to distinguish the isomers in this study. (*Z*)-3-hexenol, methyl phenylacetate, and geraniol were identified in control and experimental filters and were hence considered contaminants and excluded from all analyses (Supplementary Information S2). Butylated hydroxytoluene was also considered a contaminant and excluded from analysis as it is not a known natural product from plants (DF, personal observation), though produced by algae (Babu and Wu 2008), it is an antioxidant found in PVC tubing as used in this study (Hill et al. 2003).

## Results

### Total emission of floral scent

The total amount of floral VOC emitted by the flowers varied by two orders of magnitude between taxa, from 18.5 to 767.9 ng min^−1^, with *S. lumholtzianum* emitting 3–42 times more VOC than other taxa (*F*_6,40_ = 17.1, *P* < 0.001; Tukey’s HSD *P* < 0.001) (Table 2, Fig. 1, Supplementary Information S3). Pollen containing flowers emitted 5–1039% (*S. grayi* ssp. *grayi* and *S. lumholtzianum*, respectively) more scent than those without pollen (F_1,40_ = 7.7, *P* < 0.01) (Fig. 1). The between-treatment difference was significant only in a single species, *S. lumholtzianum* (interaction: *F*_1,40_ = 9.5, *P* < 0.001; *t* test: *t*_6_ = 7.9, *P* < 0.05) (Supplementary Information S5), and not between or within any other taxa (Fig. 1).

**Table 2:** Total floral volatile organic compound emission per flower of taxa in *Solanum* section *Androceras* with and without pollen, and mean emission of individual chemical compounds (ng min^−1^ flower^−1^)

	*S. citrullifolium*		*S. heterodoxum*		*S. rostratum*		*S. fructu-tecto*		*S. g. grandiflorum*		*S. g. grayi*		*S. lumholtzianum*	
Sample size (*n*)	4		4		4		4		4		4		3	
Treatment	+ Pollen	– Pollen	+ Pollen	– Pollen	+ Pollen	– Pollen	+ Pollen	-Pollen	+ Pollen	– Pollen	+ Pollen	– Pollen	+ Pollen	– Pollen
Total emission (ng min^−1^ flower^−1^)	259.3	51.9	96.4	135.4	43.1	18.5	147.3	121.5	142.5	38.1	197.4	188.1	767.9	67.4
	(145.6)	(31.2)	(68.5)	(100.8)	(44.4)	(9.2)	(71.2)	(147.1)	(138.3)	(22.1)	(114.5)	(28.2)	(120.9)	(33.4)
Z3-hexenyl acetate	42.4	4.1	7.9	24.0	6.6	1.7	–	–	11.0	2.7	75.2	36.5	12.9	9.8
	(26.6)	(7.0)	(13.8)	(33.0)	(5.1)	(3.0)			(10.5)	(4.6)	(60.9)	(7.7)	(18.3)	(13.8)
Linalool	4.0	–	–	–	–	–	–	–	3.3	–	–	–	498.1	–
	(4.9)								(5.7)				(126.8)	
Caryophyllene	26.2	1.4	15.9	12.5	–	–	11.3	–	86.4	11.9	38.1	52.3	–	–
	(22.5)	(2.4)	(27.5)	(21.6)			(19.5)		(102.4)	(20.7)	(25.1)	(6.3)		
Farnesal	44.0	34.8	54.8	78.7	11.1	14.4	110.8	87.4	20.0	10.4	49.7	64.3	85.6	52.9
	(33.7)	(19.4)	(56.2)	(86.4)	(8.2)	(11.6)	(58.1)	(105.8)	(14.7)	(6.0)	(35.0)	(30.2)	(54.2)	(15.7)
Farnesol	142.7	11.7	17.7	20.3	25.3	2.3	25.3	34.2	21.8	13.2	34.3	35.1	171.2	4.7
	(92.6)	(7.1)	(30.7)	(12.6)	(41.4)	(2.5)	(25.9)	(41.2)	(13.0)	(13.5)	(29.6)	(37.3)	(140.5)	(6.7)

Standard deviation presented in brackets ( ±)

### Composition of floral scent

**Fig. 2:**
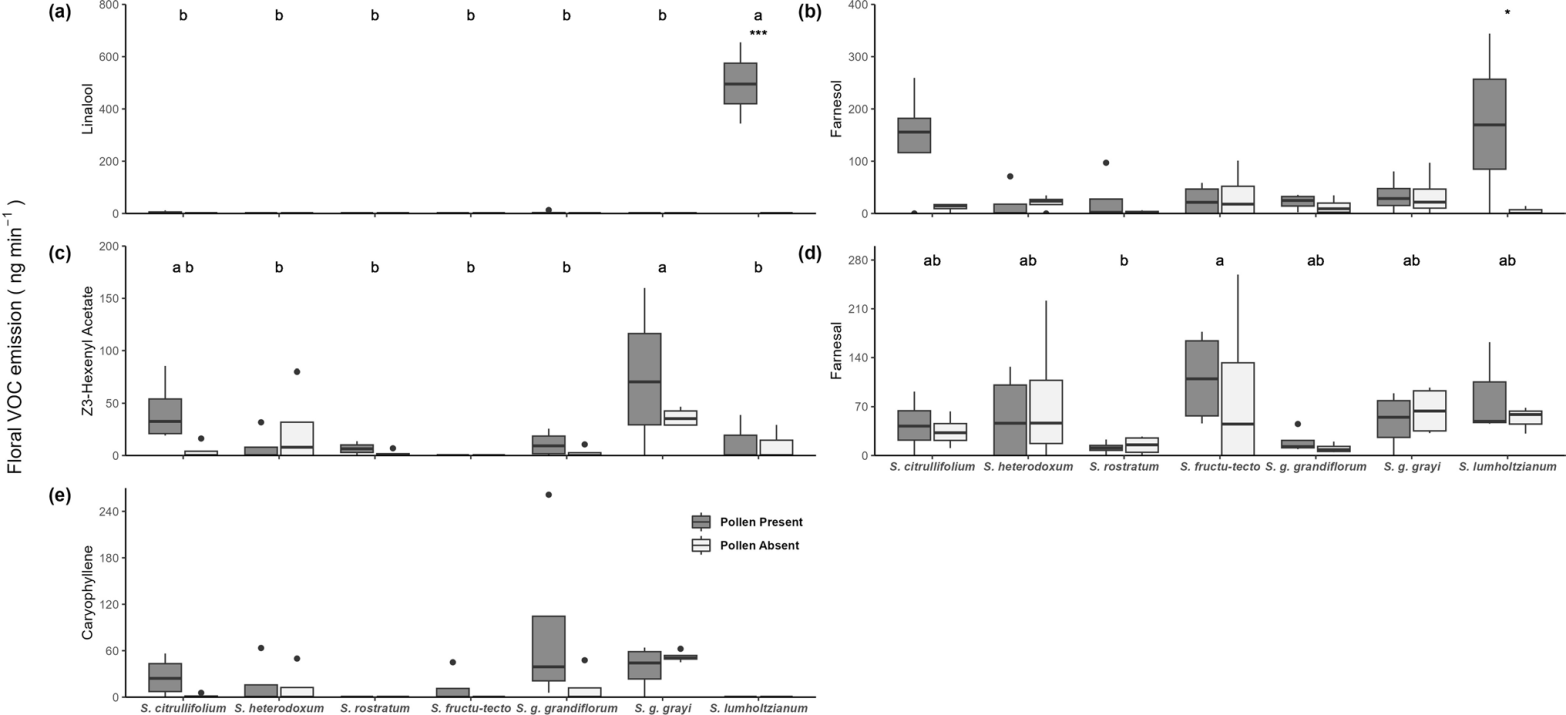
Boxplot of five VOC emitted by *Solanum* section *Androceras* flowers with and without pollen. Linalool **a** was detected only in pollen-containing flowers of *S. citrullifolium*, *S. rostratum* and *S. lumholtzianum*. *Solanum lumholtzianum* emitted more linalool than all other taxa which decreased significantly after removal of pollen. Farnesol emission **b** in *S. lumholtzianum* was also significantly higher in pollen-containing flowers. Significant between-species differences were detected in the floral emission of hexenyl acetate and farnesal (**c** and **d**, respectively) but not in caryophyllene (**e**). Asterisks denote within-species statistically significant differences (*P < 0.05, ***P < 0.001) and letters denote between-species differences (*P* < 0.05)

**Fig. 3:**
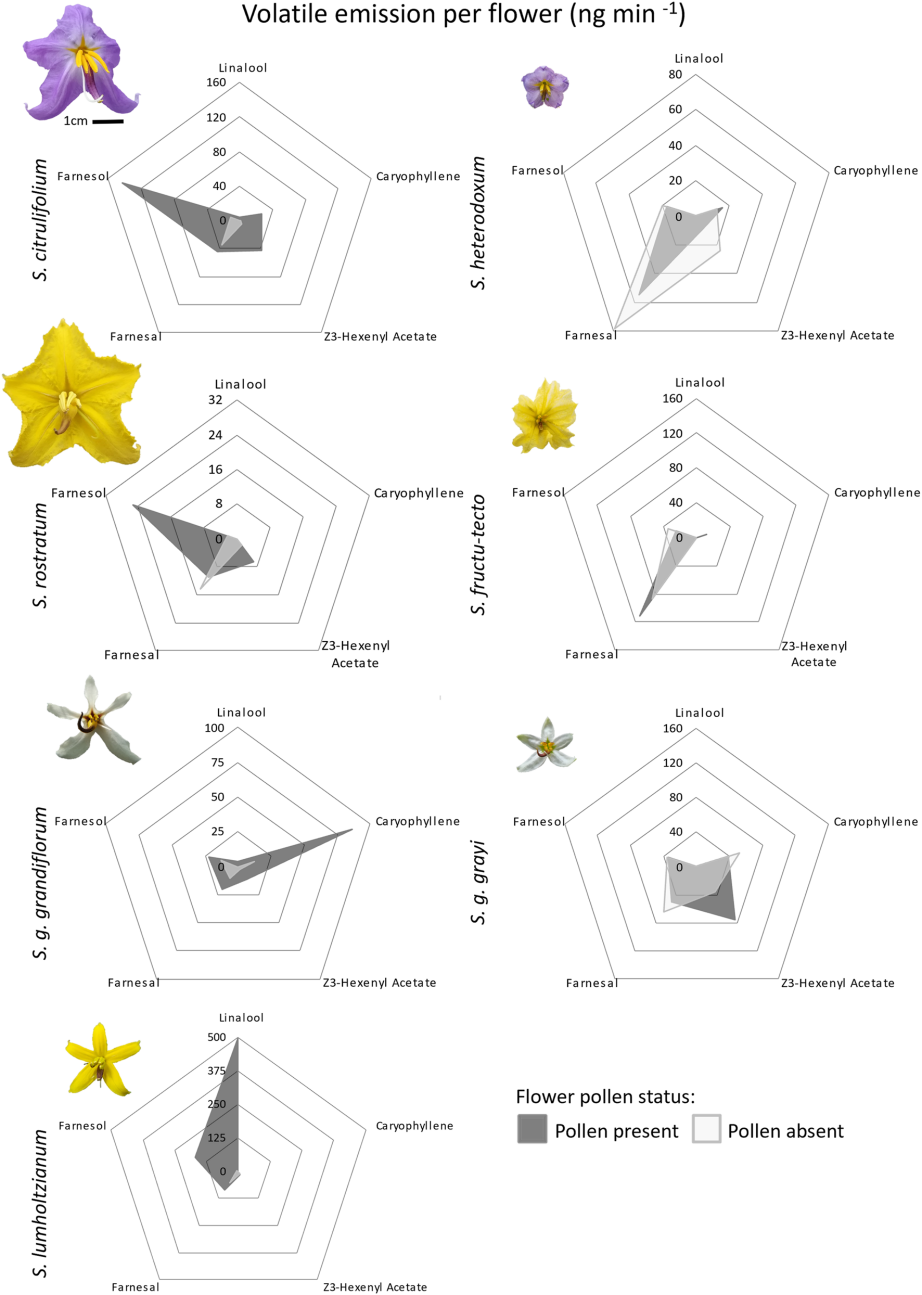
Radar plots of floral scent composition for each of the seven studied taxa of *Solanum* section *Androceras*. Floral scent composition varied between taxa and changed after pollen was removed. Linalool was emitted by pollen-containing flowers of *S. lumholtzianum*, *S. grayi* ssp. *grandiflorum* and *S. citrullifolium* but was not detected in the headspace of any flowers without pollen. Linalool and farnesol emission in *S. lumholtzianum* reduced significantly upon pollen removal

Five VOC detected in the floral headspace in *Solanum* section *Androceras* varied significantly between taxa (MANOVA: *F*_4,40_ = 4.03, *P* < 0.001) (Figs. 2, 3, Table 1, Supplementary Information S6). Two of these, linalool and farnesol, also reduced in response to pollen removal (*P* < 0.001), with significant differences in the response to pollen removal detected between taxa (interaction *P* < 0.001) (Fig. 3; Supplementary Information S3; Supplementary Information S4). Linalool was only detected in the headspace of pollen-containing flowers of the three large-displaying taxa, *S. lumholtzianum*, *S. grayi* ssp. *grandiflorum*, and *S. citrullifolium*, where it comprised 64.5%, 0.9%, and 1.3% of the with-pollen floral scent, respectively (Table 2). *Solanum lumholtzianum* emitted significantly more linalool than other taxa (taxon: *F*_6,40_ = 45.28, *P* < 0.001; Tukey’s Pairwise HSD: *P* < 0.001) (Fig. 2a), where linalool emissions responded dramatically to pollen removal decreasing from 498.1 to 0 ng min^−1^ (treatment: *F* = 35.45, *P* < 0.001; Tukey’s pairwise HSD: *P* < 0.001), and was responsible for the significant interaction (*F* = 45.28, *P* < 0.001; Tukey’s Pairwise HSD: *P* < 0.001). Linalool was also detected only in pollen-containing flowers of *S. grayi* ssp. *grandiflorum* and *S. citrullifolium* (i.e. linalool was not detected in flowers without pollen), but emissions were low and the change was not statistically significant (Table 2). Farnesol was detected in all taxa and varied significantly with pollen presence (*F*_6,40_ = 7.182, *P* < 0.05) (Fig. 2b, Table 2), with the greatest difference observed in *S. lumholtzianum* where pollen-containing flowers emitted 36 times more farnesol than those without (171 ng min^−1^ and 5 ng min^−1^ respectively; Tukey’s Pairwise HSD, *P* < 0.05) (Figs. 2, 3). There were no other significant differences between or within species; however, the interaction was significant (*F*_6,40_ = 2.9202, *P* < 0.05).

Three further VOC showed significant differences between species but not associated with pollen presence. Hexenyl acetate was detected in all taxa except *S. fructu-tecto* and varied significantly between taxa (*F*_6,40_ = 4.463, *P* < 0.005) (Fig. 2c, Table 2). Hexenyl acetate accounted for 24.6% of the floral scent in *S. grayi* ssp. *grayi* (55.9 ng^−1^ min^−1^), significantly more than in all other taxa (Tukey’s Pairwise HSD, *P* < 0.05) except *S. citrullifolium* (mean 23.3 ng^−1^ min^−1^). Farnesal was detected in all taxa with significant differences in emission between species: *S. fructu-tecto* produced 99.1 ng^−1^ min^−1^, 7.8 times more than *S. rostratum* (*F*_6,40_ = 2.409, *P* < 0.05) (Fig. 2d, Table 2). Caryophyllene was recorded in all taxa except *S. rostratum* and *S. lumholtzianum* and varied significantly between taxa (*F*_6,40_ = 2.390, *P* < 0.05) (Fig. 2e, Table 2) although no pairwise comparisons were significant (Tukey’s pairwise HSD *P* > 0.05).

## Discussion

We set out to address whether floral scents vary depending upon the availability of visually concealed pollen, which could be used by foraging pollinators. Specifically, we asked whether seven poricidal taxa change their floral scent upon pollen removal. Species of *Solanum* section *Androceras* have long been known to be chemically distinct due to the evolution of novel flavonoid biosynthetic pathways during the diversification of the group (Whalen 1978). It is therefore unsurprising that between-species differences were found in three chemicals detected in this study: linalool, hexenyl acetate, and farnesal. Of far greater consequence was our finding that vibrational pollen removal causes significant changes in *Solanum lumholtzianum* VOC, with reductions in the emission of total floral scent and of individual chemicals, namely linalool and farnesol. Indeed, linalool was only detected in the headspace of flowers containing pollen, present also in *S. citrullifolium* and *S. grayi* ssp. *grandiflorum*, but absent in flowers without pollen. Other authors have described correlations between floral traits and nectar (Knauer and Schiestl 2015; Gervasi and Schiestl 2017; Parachnowitsch et al. 2019), but this is the first demonstration of rewards and signals correlating in pollen rewarding plants. Yet, detecting reward-associated scent variation in one of seven taxa suggests that this may be a rare trait, and that buzz-pollinated flowers more commonly conceal their reward status from pollinators not only visually but also through their scent.

Signals and cues which correlate with reward quality or quantity can be considered honest as they provide accurate information on the status of rewards (van der Kooi et al. 2023). We demonstrated that VOC emission correlated with pollen presence in one species, *S. lumholtzianum*. Indeed, that linalool was emitted by pollen-containing flowers of two further taxa suggests a conserved mechanism for honest signalling may exist in the group, but this needs further investigation. Honest reward signals are expected to evolve and persist where they are beneficial, by increasing plant export and receipt of pollen, and will be used where they improve the efficiency of pollinator learning, handling, and profitability (Dobson and Bergström, 2000; Raguso 2004). Honest signalling is expected to break down when these conditions are not met or are rendered irrelevant by plant transitions away from scent-oriented pollinators or zoophily entirely (Gervasi and Schiestl 2017). Such changes in reproductive strategies have been suggested in section *Androceras* (Vallejo-Marín et al. 2014) which may explain why significant scent changes were not observed in response to pollen removal in all taxa examined. We would therefore not expect all species with pollen-only flowers to present honest scent signals, rather those which rely most heavily on scent-oriented pollinators such as bees.

Scented pollen is produced by many angiosperm taxa, contributing to flower scent and pollinator attraction (Dobson et al. 1999; Muth et al. 2016). Thus, we hypothesised that pollen is a source of some floral VOC in *S. lumholtzianum*, the removal of which changes overall flower scent. Yet, we did not detect any pollen emitted VOC. Indeed, the contribution of pollen volatiles to floral scent is generally understudied and the few studies conducted on poricidal flowers have produced equivocal results. Pollen-associated VOC were reported in some *Solanum* species (Kessler and Halitschke 2009; Palmer-Young et al. 2019) but not in others (this study; Solís-Montero et al. 2018). This could be due to pollen scent being a highly variable, species-specific trait, or due to methodological differences (e.g. solvent choice, extraction duration, sample size, or sampling techniques). However, pollen of buzz-pollinated flowers may be unscented. In most angiosperms, pollen scent is emitted by a pollen grain coating: pollenkitt (Pacini and Hesse 2005). Yet, as pollenkitt is adhesive, causing pollen grain clumping, it is greatly reduced or absent in plants which rely on airborne pollen transfer as in wind- and buzz-pollination (Buchmann et al. 1983; Timerman and Barrett 2021). Furthermore, the internal chambers and small anther pore apertures of poricidal flowers (Buchmann and Cane 1989) could conceivably restrict scent emission and so reduce the advantage of scented pollen. Indeed, the emission of scent from osmophores on the exterior of *S. rostratum* anthers conforms to this hypothesis (Solís-Montero et al. 2018). Therefore, if pollen is unscented and visually concealed within anthers, pollinators foraging on plants which provide honest signals, like *S. lumholtzianum*, must rely entirely on cues produced by other plant tissues. However, in other taxa where flower visual and olfactory signalling are constant, pollinators can only assess pollen presence by buzzing flowers. This could improve reproductive success by increasing visitation, thus raising the probability of pollen receipt and export. If this conveyed a large reproductive advantage it could partly explain the abundance, diversity, and convergent evolution of buzz-pollinated plants. Future work across multiple buzz-pollinated flower families using a combination of standardised collection methods (e.g. solvent extraction, solid-phase microextraction, and headspace collection) would help resolve whether scented pollen is common and identify other sources of floral scent. Ideally, such investigations would include species with scented pollen as a positive control.

Alternatively, scent emission may change in response to pollen removal by insect vibrations or experimental extraction and trigger changes in plant physiology. Plants respond to environmental stimuli and damage (e.g. herbivory) by producing secondary metabolites which alter plant physiology or signal to conspecifics and natural allies (Kessler et al. 2011; Atamian et al. 2016). Such responses can be localised or systemic. Physiological responses to herbivory can reduce pollinator attraction by upregulating repellent compounds and downregulating attractive ones (Kessler and Halitschke 2009; Kessler et al. 2011). Furthermore, florivory causes floral scent change in *S. rostratum*, reducing pollinator and herbivore attraction but not reproductive success (Vega-Polanco et al. 2020). Indeed, the removal of pollen, pollinaria, or nectar without pollination may trigger flower senescence (Richardson and Stephenson 1989; Clayton and Aizen 1996; Huda and Wilcock 2012) similar to post-pollination responses (Theis and Raguso 2005; Herrera 2011). This suggests that plants may detect the removal of floral rewards by mechanisms other than damage which may explain the observed scent changes in *S. lumholtzianum*. Further investigation to disentangle the mechanism of VOC change, could compare scent of flowers from which pollen has been extracted by buzzing bees, by damaging and non-damaging experimental methods (i.e. sound waves or indirect vibrations: Kemp and Vallejo-Marín 2021; Nunes et al. 2021) and by vibrating anthers without extracting pollen accompanied by a comprehensive investigation of whether pollen emits VOC directly. However, regardless of the mechanism which causes scent change, bees will use perceptible differences between flowers to inform foraging decisions (Rains et al. 2008; Clarke et al. 2013; Harrap et al. 2020). This might, therefore, represent an important but overlooked mode of plant–pollinator signalling in poricidal flowers worthy of further investigation, especially in relation to pollinator foraging behaviour.

Our study suggests that floral scents associated with reward (i.e. pollen) could represent an example of honest signalling used by flowers with concealed rewards and in pollen-only flowers. We showed that headspace of the pollen-only, poricidal flowers of *S. lumholtzianum* change in response to pollen removal, although more work is required to determine whether this is attributed to the loss of pollen emitted VOC. Emission of both linalool, a chemical with well-characterised pollinator attractive function, and farnesol positively correlated with pollen availability, and could be used by pollinators when making foraging decisions. Yet, the six other taxa studied exhibited no significant VOC change, suggesting that buzz-pollinated flowers commonly prevent remote reward assessment using scent as they do to sight.

### Supplementary Information

Below is the link to the electronic supplementary material.

Supplementary file1 (DOCX 579 KB)

## Data Availability

The dataset generated and analysed in this study is available in DataStorre: http://hdl.handle.net/11667/209.

## Abbreviation

VOC: Volatile organic compound

## References

[CR1] Atamian HS, Creux NM, Brown EA (2016). Circadian regulation of sunflower heliotropism, floral orientation, and pollinator visits. Science.

[CR2] Austin MW, Horack P, Dunlap AS (2019). Choice in a floral marketplace: the role of complexity in bumble bee decision-making. Behav Ecol.

[CR3] Babu B, Wu JT (2008). Production of natural butylated hydroxytoluene as an antioxidant by freshwater phytoplankton. J Phycol.

[CR4] Buchmann SL, Cane JH (1989). Bees assess pollen returns while sonicating *Solanum* flowers. Oecologia.

[CR5] Buchmann SL, Jones CE, Little RJ, Jones CE, Little RJ (1983). Buzz pollination in angiosperms. Handbook of experimental pollination biology.

[CR6] Burdon RCF, Raguso RA, Kessler A, Parachnowitsch AL (2015). Spatiotemporal floral scent variation of *Penstemon digitalis*. J Chem Ecol.

[CR7] Burdon RCF, Raguso RA, Gegear RJ (2020). Scented nectar and the challenge of measuring honest signals in pollination. J Ecol.

[CR8] Byers KJRP, Bradshaw HD, Riffell JA (2014). Three floral volatiles contribute to differential pollinator attraction in monkeyflowers (*Mimulus*). J Exp Biol.

[CR9] Cardinal S, Buchmann SL, Russell AL (2018). The evolution of floral sonication, a pollen foraging behavior used by bees (Anthophila). Evolution.

[CR10] Clarke D, Whitney H, Sutton G, Robert D (2013). Detection and learning of floral electric fields by bumblebees. Science.

[CR11] Clayton S, Aizen MA (1996). Effects of pollinia removal and insertion on flower longevity in *Chloraea alpina* (Orchidaceae). Evol Ecol.

[CR12] Crepet WL, Real L (1983). The role of insect pollination in the evolution of angiosperms. Pollination biology.

[CR13] De Luca PA, Vallejo-Marín M (2013). What’s the “buzz” about? The ecology and evolutionary significance of buzz-pollination. Curr Opin Plant Biol.

[CR14] Dobson HEM, Bernays EA (1994). Floral volatiles in insect biology. Insect-plant interactions.

[CR15] Dobson HEM, Bergström G (2000). The ecology and evolution of pollen odors. Plant Syst Evol.

[CR16] Dobson HEM, Danielson EM, Van Wesep ID (1999). Pollen odor chemicals as modulators of bumble bee foraging on *Rosa rugosa* Thunb. (Rosaceae). Plant Species Biol.

[CR17] Dötterl S, Jürgens A (2005). Spatial fragrance patterns in flowers of *Silene latifolia*: lilac compounds as olfactory nectar guides?. Plant Syst Evol.

[CR18] Farré-Armengol G, Filella I, Llusià J (2014). Changes in floral bouquets from compound-specific responses to increasing temperatures. Glob Chang Biol.

[CR19] Filella I, Primante C, Llusià J (2013). Floral advertisement scent in a changing plant-pollinators market. Sci Rep.

[CR20] Fudge DS, Turko AJ (2020). The best predictions in experimental biology are critical and persuasive. J Exp Biol.

[CR21] García Y, Friberg M, Parachnowitsch AL (2021). Spatial variation in scent emission within flowers. Nord J Bot.

[CR22] Gervasi DDL, Schiestl FP (2017). Real-time divergent evolution in plants driven by pollinators. Nat Commun.

[CR23] Gfrerer E, Laina D, Gibernau M (2021). Floral scents of a deceptive plant are hyperdiverse and under population-specific phenotypic selection. Front Plant Sci.

[CR24] Harrap MJM, Hempel de Ibarra N, Whitney HM, Rands SA (2020). Floral temperature patterns can function as floral guides. Arthropod Plant Interact.

[CR25] Heard SB (2016). The scientist’s guide to writing: how to write more easily and effectively throughout your scientific career.

[CR26] Herrera CM (2011). Complex implications around a simple trait: ecological context determines the fecundity effects of corolla marcescence. Am J Bot.

[CR27] Hill SS, Shaw BR, Wu AHB (2003). Plasticizers, antioxidants, and other contaminants found in air delivered by PVC tubing used in respiratory therapy. Biomed Chromatogr.

[CR28] Hotaling S (2020). Simple rules for concise scientific writing. Limnol Oceanogr Lett.

[CR29] Huang M, Sanchez-Moreiras AM, Abel C (2012). The major volatile organic compound emitted from *Arabidopsis thaliana* flowers, the sesquiterpene (E)-β-caryophyllene, is a defense against a bacterial pathogen. New Phytol.

[CR30] Huda MK, Wilcock CC (2012). Rapid floral senescence following male function and breeding systems of some tropical orchids. Plant Biol.

[CR31] Jacquemart AL, Buyens C, Hérent MF (2019). Male flowers of *Aconitum* compensate for toxic pollen with increased floral signals and rewards for pollinators. Sci Rep.

[CR32] Jaworski CC, Geslin B, Zakardjian M (2022). Long-term experimental drought alters floral scent and pollinator visits in a Mediterranean plant community despite overall limited impacts on plant phenotype and reproduction. J Ecol.

[CR33] Kemp JE, Vallejo-Marín M (2021). Pollen dispensing schedules in buzz-pollinated plants: experimental comparison of species with contrasting floral morphologies. Am J Bot.

[CR34] Kessler A, Halitschke R (2009). Testing the potential for conflicting selection on floral chemical traits by pollinators and herbivores: predictions and case study. Funct Ecol.

[CR35] Kessler A, Halitschke R, Poveda K (2011). Herbivory-mediated pollinator limitation: negative impacts of induced volatiles on plant-pollinator interactions. Ecology.

[CR36] King MJ, Buchmann SL (1996). Sonication dispensing of pollen from *Solanum laciniatum* flowers. Funct Ecol.

[CR37] Knapp S, Cronk QCB, Bateman RM, Hawkins JA (2002). Floral diversity and evolution in the Solanaceae. Developmental genetics and plant evolution.

[CR38] Knauer AC, Schiestl FP (2015). Bees use honest floral signals as indicators of reward when visiting flowers. Ecol Lett.

[CR39] Knudsen ER, Gershenzon J, Stahl B (2006). Diversity and distribution of floral scent. Bot Rev.

[CR40] Leonard AS, Dornhaus A, Papaj DR, Patiny S (2011). Why are floral signals complex? An outline of functional hypotheses. Evolution of plant-pollinator relationships.

[CR41] Morinaga SI, Kumano Y, Ota A (2009). Day-night fluctuations in floral scent and their effects on reproductive success in *Lilium auratum*. Popul Ecol.

[CR42] Muhlemann JK, Klempien A, Dudareva N (2014). Floral volatiles: from biosynthesis to function. Plant Cell Environ.

[CR43] Muth F, Papaj DR, Leonard AS (2016). Bees remember flowers for more than one reason: pollen mediates associative learning. Anim Behav.

[CR44] Nunes CEP, Nevard L, Montealegre-Z F, Vallejo-Marín M (2021). Variation in the natural frequency of stamens in six morphologically diverse, buzz-pollinated, heterantherous *Solanum* taxa and its relationship to bee vibrations. Bot J Linn Soc.

[CR45] Pacini E, Hesse M (2005). Pollenkitt - its composition, forms and functions. Flora Morphol Distrib Funct Ecol Plants.

[CR46] Palmer-Young EC, Farrell IW, Adler LS (2019). Chemistry of floral rewards: intra- and interspecific variability of nectar and pollen secondary metabolites across taxa. Ecol Monogr.

[CR47] Parachnowitsch AL, Manson JS, Sletvold N (2019). Evolutionary ecology of nectar. Ann Bot.

[CR48] Raguso RA (2004). Why are some floral nectars scented?. Ecology.

[CR49] Raguso RA, Pichersky E (1999). A day in the life of a linalool molecule: chemical communication in a plant-pollinator system. Part 1: linalool biosynthesis in flowering plants. Plant Species Biol.

[CR50] Raguso RA, Willis MA (2005). Synergy between visual and olfactory cues in nectar feeding by wild hawkmoths, *Manduca sexta*. Anim Behav.

[CR51] Rains GC, Tomberlin JK, Kulasiri D (2008). Using insect sniffing devices for detection. Trends Biotechnol.

[CR52] Richardson TE, Stephenson AG (1989). Pollen removal and pollen deposition affect the duration of the staminate and pistillate phases in *Campanula rapunculoides*. Am J Bot.

[CR53] Russell AL, Leonard AS, Gillette HD, Papaj DR (2016). Concealed floral rewards and the role of experience in floral sonication by bees. Anim Behav.

[CR54] Russell AL, Buchmann SL, Papaj DR (2017). How a generalist bee achieves high efficiency of pollen collection on diverse floral resources. Behav Ecol.

[CR55] Russell AL, Mauerman KB, Golden RE, Papaj DR (2018). Linking components of complex signals to morphological part: the role of anther and corolla in the complex floral display. Anim Behav.

[CR56] Şanli Ö, Erdem S, Tefik T (2013). How to write a discussion section?. Turk J Urol.

[CR57] Särkinen T, Bohs L, Olmstead RG, Knapp S (2013). A phylogenetic framework for evolutionary study of the nightshades (Solanaceae): a dated 1000-tip tree. BMC Evol Biol.

[CR58] Scala A, Allmann S, Mirabella R (2013). Green leaf volatiles: a plant’s multifunctional weapon against herbivores and pathogens. Int J Mol Sci.

[CR59] Schiestl FP, Peakall R, Mant JG (2003). The chemistry of sexual deception in an orchid-wasp pollination system. Science.

[CR60] Silva-Neto CM, Bergamini LL, Elias MAS (2016). High species richness of native pollinators in Brazilian tomato crops. Braz J Biol.

[CR61] Solís-Montero L, Vergara CH, Vallejo-Marín M (2015). High incidence of pollen theft in natural populations of a buzz-pollinated plant. Arthropod Plant Interact.

[CR62] Solís-Montero L, Cáceres-García S, Alavez-Rosas D (2018). Pollinator preferences for floral volatiles emitted by dimorphic anthers of a buzz-pollinated herb. J Chem Ecol.

[CR63] Stern SR, Weese T, Bohs LA (2010). Phylogenetic relationships in *Solanum* section *Androceras* (Solanaceae). Syst Bot.

[CR64] Stevenson PC, Nicolson SW, Wright GA (2017). Plant secondary metabolites in nectar: impacts on pollinators and ecological functions. Funct Ecol.

[CR65] Stewart-Jones A, Poppy GM (2006). Comparison of glass vessels and plastic bags for enclosing living plant parts for headspace analysis. J Chem Ecol.

[CR66] Theis N, Raguso RA (2005). The effect of pollination on floral fragrance in thistles. J Chem Ecol.

[CR67] Timerman D, Barrett SCH (2021). The biomechanics of pollen release: new perspectives on the evolution of wind pollination in angiosperms. Biol Rev.

[CR68] Vallejo-Marín M, Walker C, Friston-Reilly P (2014). Recurrent modification of floral morphology in heterantherous *Solanum* reveals a parallel shift in reproductive strategy. Philos Trans R Soc B Biol Sci.

[CR69] van der Kooi CJ, Reuvers L, Spaethe J (2023). Honesty, reliability, and information content of floral signals. iScience.

[CR70] Vega-Polanco M, Rodríguez-Islas LA, Escalona-Domenech RY (2020). Does florivory affect the attraction of floral visitors to buzz-pollinated *Solanum rostratum*?. Arthropod Plant Interact.

[CR71] Westerkamp C (1997). Keel blossoms: bee flowers with adaptations against bees. Flora.

[CR72] Westerkamp C, Claßen-Bockhoff R (2007). Bilabiate flowers: the ultimate response to bees?. Ann Bot.

[CR73] Whalen MD (1978). Foliar flavonoids of *Solanum* section *Androceras*: a systematic survey. Syst Bot.

[CR74] Wickham H (2016). Package “ggplot2”: elegant graphics for data analysis.

[CR75] Wright GA, Schiestl FP (2009). The evolution of floral scent: the influence of olfactory learning by insect pollinators on the honest signalling of floral rewards. Funct Ecol.

[CR76] Yu JS, Kleckley TS, Wiemer DF (2005). Synthesis of farnesol isomers via a modified wittig procedure. Org Lett.

[CR77] Zhao Z, Zung JL, Hinze A (2022). Mosquito brains encode unique features of human odour to drive host seeking. Nature.

[CR78] El-Sayed AM (2019) The pherobase: database of pheromones and semiochemicals. https://www.pherobase.com/. Accessed 25 Nov 2021

[CR79] R Core Team (2022) R: a language and environment for statistical computing

