# Supplementary material for: Floral scent changes in response to pollen removal are rare in buzz-pollinated Solanum
Source: Planta. 2024 Jun 3;260(1):15. doi: 10.1007/s00425-024-04403-4 (PMC11147924; doi:10.1007/s00425-024-04403-4)
Supplement: Supplementary file 1 — Supplementary file1 (DOCX 579 KB) [file 425_2024_4403_MOESM1_ESM.docx]

***Supplementary Information***

Floral scent changes in response to pollen removal are rare in buzz-pollinated *Solanum*

*Planta*

**C. Douglas Moore1*, Dudley I. Farman, Tiina Särkinen, Philip C. Stevenson, and Mario Vallejo-Marín**

*** Correspondence:** C. Douglas Moore: [c.d.moore@stir.ac.uk](mailto:c.d.moore@stir.ac.uk), Orchid-ID: 0000-0003-3648-4273

^1^Biological and Environmental Sciences, University of Stirling, Stirling FK9 4LA, United Kingdom

**Supplementary Information S1** Headspace samples were collected from all open flowers per plant giving a variable number of flowers per collection

| **Taxon** | **Treatment** | **Replicate** | **Number of Flowers** |
| --- | --- | --- | --- |
| ***S. citrullifolium*** | Pollen Present | 1 | 8 |
|  |  | 2 | 5 |
|  |  | 3 | 11 |
|  |  | 4 | 15 |
|  | Pollen Absent | 1 | 12 |
|  |  | 2 | 16 |
|  |  | 3 | 11 |
|  |  | 4 | 6 |
| ***S. heterodoxum*** | Pollen Present | 1 | 3 |
|  |  | 2 | 4 |
|  |  | 3 | 3 |
|  |  | 4 | 5 |
|  | Pollen Absent | 1 | 7 |
|  |  | 2 | 5 |
|  |  | 3 | 4 |
|  |  | 4 | 4 |
| ***S. rostratum*** | Pollen Present | 1 | 13 |
|  |  | 2 | 19 |
|  |  | 3 | 8 |
|  |  | 4 | 7 |
|  | Pollen Absent | 1 | 20 |
|  |  | 2 | 6 |
|  |  | 3 | 12 |
|  |  | 4 | 9 |
| ***S. fructu-tecto*** | Pollen Present | 1 | 1 |
|  |  | 2 | 3 |
|  |  | 3 | 1 |
|  |  | 4 | 3 |
|  | Pollen Absent | 1 | 2 |
|  |  | 2 | 2 |
|  |  | 3 | 2 |
|  |  | 4 | 2 |
| ***S. g. grandiflorum*** | Pollen Present | 1 | 25 |
|  |  | 2 | 16 |
|  |  | 3 | 21 |
|  |  | 4 | 20 |
|  | Pollen Absent | 1 | 20 |
|  |  | 2 | 15 |
|  |  | 3 | 17 |
|  |  | 4 | 11 |
| ***S. g. grayi*** | Pollen Present | 1 | 6 |
|  |  | 2 | 5 |
|  |  | 3 | 6 |
|  |  | 4 | 2 |
|  | Pollen Absent | 1 | 9 |
|  |  | 2 | 3 |
|  |  | 3 | 3 |
|  |  | 4 | 2 |
| ***S. lumholtzianum*** | Pollen Present | 1 | 3 |
|  |  | 2 | 1 |
|  |  | 3 | 4 |
|  | Pollen Absent | 1 | 4 |
|  |  | 2 | 1 |
|  |  | 3 | 5 |

**Supplementary Information S2** Mean emission of all volatiles detected in the headspace of *Solanum* section *Androceras* (ng min^-1^ flower^-1^). Contaminant VOC eliminated from analyses (*) and SD

|  | ***S. citrullifolium*** | | ***S. heterodoxum*** | | ***S. rostratum*** | | ***S. fructu-tecto*** | | ***S. grayi* ssp.**  ***grandiflorum*** | | ***S. grayi* ssp.**  ***grayi*** | | ***S. lumholtzianum*** | |
| --- | --- | --- | --- | --- | --- | --- | --- | --- | --- | --- | --- | --- | --- | --- |
| Sample size (*n*) | 4 | | 4 | | 4 | | 4 | | 4 | | 4 | | 3 | |
| Treatment | +Pollen | -Pollen | +Pollen | -Pollen | +Pollen | -Pollen | +Pollen | -Pollen | +Pollen | -Pollen | +Pollen | -Pollen | +Pollen | -Pollen |
| Total Emission (ng min^-1^ flower^-1^) | 588.6 | 177.0 | 390.2 | 354.3 | 112.3 | 75.7 | 605.1 | 501.6 | 270.0 | 101.1 | 537.1 | 534.6 | 2388.0 | 476.8 |
|  | 347.9 | 159.4 | 283.9 | 242.0 | 64.5 | 35.1 | 218.8 | 348.7 | 249.7 | 24.9 | 308.7 | 235.2 | 314.9 | 172.8 |
| Z3-Hexenyl Acetate | 42.4 | 4.1 | 7.9 | 24.0 | 6.6 | 1.7 | 0.0 | 0.0 | 11.0 | 2.7 | 75.2 | 36.5 | 12.9 | 9.8 |
|  | 26.6 | 7.0 | 13.8 | 33.0 | 5.1 | 3.0 | 0.0 | 0.0 | 10.5 | 4.6 | 60.9 | 7.7 | 18.3 | 13.8 |
| Z3-Hexenol* | 14.0 | 18.3 | 30.6 | 17.3 | 12.0 | 11.7 | 144.5 | 60.5 | 17.1 | 9.4 | 21.6 | 36.0 | 88.8 | 61.0 |
|  | 6.9 | 11.2 | 8.7 | 3.1 | 11.6 | 0.7 | 119.9 | 14.9 | 10.9 | 7.0 | 7.9 | 16.5 | 59.0 | 28.0 |
| Linalool | 4.0 | 0.0 | 0.0 | 0.0 | 0.0 | 0.0 | 0.0 | 0.0 | 3.3 | 0.0 | 0.0 | 0.0 | 498.1 | 0.0 |
|  | 4.9 | 0.0 | 0.0 | 0.0 | 0.0 | 0.0 | 0.0 | 0.0 | 5.7 | 0.0 | 0.0 | 0.0 | 126.8 | 0.0 |
| Caryophyllene | 26.2 | 1.4 | 15.9 | 12.5 | 0.0 | 0.0 | 11.3 | 0.0 | 86.4 | 11.9 | 38.1 | 52.3 | 0.0 | 0.0 |
|  | 22.5 | 2.4 | 27.5 | 21.6 | 0.0 | 0.0 | 19.5 | 0.0 | 102.4 | 20.7 | 25.1 | 6.3 | 0.0 | 0.0 |
| Methyl Phenylacetate* | 51.6 | 3.9 | 62.9 | 27.2 | 21.2 | 13.3 | 50.1 | 43.9 | 13.8 | 8.9 | 21.6 | 54.2 | 164.1 | 33.4 |
|  | 30.5 | 4.0 | 57.1 | 31.8 | 27.6 | 16.4 | 21.4 | 21.1 | 10.2 | 8.0 | 24.1 | 47.4 | 35.9 | 16.3 |
| Geraniol* | 189.7 | 11.2 | 42.1 | 76.2 | 14.5 | 13.6 | 182.4 | 134.6 | 30.7 | 8.2 | 196.4 | 126.0 | 999.9 | 83.9 |
|  | 133.5 | 7.0 | 29.6 | 69.4 | 9.0 | 7.5 | 125.3 | 50.9 | 18.3 | 4.1 | 130.3 | 148.8 | 338.4 | 39.2 |
| Butylated hydroxytoluene* | 73.8 | 91.7 | 158.2 | 98.1 | 21.5 | 18.7 | 80.8 | 141.1 | 65.9 | 36.4 | 100.0 | 130.2 | 367.3 | 231.1 |
|  | 77.5 | 126.5 | 158.6 | 99.4 | 37.2 | 32.4 | 140.0 | 244.4 | 86.3 | 37.1 | 101.4 | 184.6 | 116.3 | 166.7 |
| Farnesal | 44.0 | 34.8 | 54.8 | 78.7 | 11.1 | 14.4 | 110.8 | 87.4 | 20.0 | 10.4 | 49.7 | 64.3 | 85.6 | 52.9 |
|  | 33.7 | 19.4 | 56.2 | 86.4 | 8.2 | 11.6 | 58.1 | 105.8 | 14.7 | 6.0 | 35.0 | 30.2 | 54.2 | 15.7 |
| Farnesol | 142.7 | 11.7 | 17.7 | 20.3 | 25.3 | 2.3 | 25.3 | 34.2 | 21.8 | 13.2 | 34.3 | 35.1 | 171.2 | 4.7 |
|  | 92.6 | 7.1 | 30.7 | 12.6 | 41.4 | 2.5 | 25.9 | 41.3 | 13.0 | 13.5 | 29.6 | 37.3 | 140.5 | 6.7 |

**Supplementary Information S3** Retention times, Kovats Indices and molecular masses of compounds emitted by *Solanum rostratum* and their identification with FID and by Solís- Montero *et al.* (2018, * indicates identities confirmed by comparing against synthetic compound). Retention times and Kovats Indices were established in this study for all volatiles below using synthetic chemicals († denotes those which were not)

| Compound | Class | Retention time | Kovats Index | Molecular weight (g/mol) | Solis Montero *et al.* (2018) | This Study |
| --- | --- | --- | --- | --- | --- | --- |
| Z3-Hexenyl Acetate | Carboxylic acid esters | 5.990 | 1294 | 142.2 |  | Yes |
| Z3-Hexenol | Fatty alcohol | 6.877 | 1364 | 100.159 |  | Yes |
| α-Copaene | Sesquiterpene | 8.134 | 1469 | 204.357 | Yes |  |
| Linalool | Acyclic monoterpenoids | 8.887 | 1533 | 154.25 |  | Yes |
| Methyl benzoate / Benzoic acid methyl ester | Benzoic acid esters | 9.689 | 1601 | 136.15 | Yes* |  |
| Ethyl benzoate  / Benzoic acid ethyl ester | Benzoic acid esters | 10.183 | 1647 | 150.177 | Yes* |  |
| (E,E)-α-  Farnesene | Sesquiterpenoids | 11.041 | 1727 | 204.351 | Yes |  |
| γ-Decalactone | Gamma butyrolactones |  |  | 170.25 | Yes* | † |
| Hexadecane | Alkanes |  |  | 226.44 | Yes* | † |
| Methyl Phenylacetate | Benzene and substituted derivatives | 11.205 | 1742 | 150.175 |  | Yes |
| Methyl salicylate | Salicylates | 11.374 | 1758 | 452.7 | Yes* |  |
| Ethyl salicylate | O-hydroxybenzoic acid esters |  |  | 166.17 | Yes* | † |

| Caryophyllene | Sesquiterpenoids | 11.857 | 1803 | 204.357 | Yes |
| --- | --- | --- | --- | --- | --- |
| Geraniol | Acyclic monoterpenoids | 12.186 | 1837 | 154.253 | Yes |
| Methyl eugenol | dimethoxybenzenes | 13.834 | 2006 | 178.23 | Yes |
| Eugenol / Methoxy eugenol | methoxyphenols | 15.169 | 2155 | 164.2 | Yes* |
| Farnesal isomer 1 | Sesquiterpenoids | 15.674 | 2213 | 220.35 | Yes |
| Farnesal isomer 2 | Sesquiterpenoids | 15.774 | 2225 | 220.35 | Yes |
| Farnesal isomer 3 | Sesquiterpenoids | 16.074 | 2261 | 220.35 | Yes |
| (E,E)-Farnesol | Sesquiterpenoids | 16.782 | 2347 | 222.37 | Yes |
| Butylated hydroxytoluene | Phenylpropanes |  |  | 220.356 | Yes† |

**Supplementary Information S4** 2-Way ANOVA with Type III corrections was used to assess the impact of plant taxa and pollen presence on total floral VOC emission (Total Floral VOC Emission ~ Pollen Presence * Plant Taxa)

|  | Sum Sq | DF | F-value | Relative *P*-Value |
| --- | --- | --- | --- | --- |
| Intercept | 269,028 | 1 | 24.2 | 1.5^-05^ *** |
| Pollen Presence | 86,022 | 1 | 7.7 | 0.0081 ** |
| Plant Taxa | 1,139,858 | 6 | 17.1 | 1.1^-09^ *** |
| Pollen Presence: Plant Taxa | 631,545 | 6 | 9.5 | 1.8e^-06^ *** |
| Residuals | 444,474 | 40 |  |  |

**Supplementary Information S5** Difference in VOC emission within taxa was tested with pairwise t-tests. Significant results are in bold

| **Plant taxon** | ***t*** | **Df** | ***P*-value** |
| --- | --- | --- | --- |
| *S. citrullifolium* | 2.41 | 3.28 | 0.088 |
| *S. heterodoxum* | -0.55 | 5.28 | 0.602 |
| *S. rostratum* | 0.94 | 3.26 | 0.412 |
| *S. fructu-tecto* | 0.27 | 4.33 | 0.797 |
| *S. g. grandiflorum* | 1.29 | 3.15 | 0.284 |
| *S. g. grayi* | 0.14 | 3.36 | 0.900 |
| *S. lumholtzianum* | 7.99 | 2.30 | **0.010** |

**Supplementary Information S6** MANOVA summary of the five VOC emitted by each taxa and the impact of pollen removal from flowers on their presence

| Summary | DF | Pillai | Approximate F | DF | Den DF | *P*-value |
| --- | --- | --- | --- | --- | --- | --- |
| Pollen Presence | 1 | 0.64805 | 13.2575 | 5 | 36 | 2.4^-07^ *** |
| Taxon | 6 | 1.88302 | 4.0274 | 30 | 200 | 1.4^-09^ *** |
| Interaction | 6 | 1.39277 | 2.5740 | 30 | 200 | 5.3^-05^ *** |
| Residuals | 40 |  |  |  |  |  |

Significance codes: 0 ‘***’ 0.001 ‘**’ 0.01 ‘*’ 0.05 ‘.’ 0.1 ‘ ’ 1

|  |  | **DF** | **Sum Sq** | **Mean Sq** | **F-value** | ***P*-value** |
| --- | --- | --- | --- | --- | --- | --- |
| **Hexenyl acetate** | **Pollen Presence** | 1 | 1743.4 | 1743.39 | 2.7406 | 0.105654 |
|  | **Taxon** | 6 | 17033.7 | 2838.94 | 4.4628 | 0.001514 ** |
|  | **Interaction** | 6 | 4915.9 | 819.32 | 1.2880 | 0.284771 |
|  | **Residuals** | 40 | 25445.4 | 636.13 |  |  |
| **Linalool** | **Pollen Presence** | 1 | 42974 | 42974 | 35.455 | 5.478e-07 *** |
|  | **Taxon** | 6 | 329307 | 54885 | 45.282 | 2.613e-16 *** |
|  | **Interaction** | 6 | 329307 | 54885 | 45.282 | 2.613e-16 *** |
|  | **Residuals** | 40 | 48483 | 1212 |  |  |
| **Caryophyllene** | **Pollen Presence** | 1 | 2950 | 2949.9 | 2.1527 | 0.15014 |
|  | **Taxon** | 6 | 19648 | 3274.6 | 2.3897 | 0.04563 * |
|  | **Interaction** | 6 | 10051 | 1675.2 | 1.2225 | 0.31521 |
|  | **Residuals** | 40 | 54813 | 1370.3 |  |  |
| **Farnesal** | **Pollen Presence** | 1 | 185 | 185.1 | 0.0584 | 0.81021 |

|  | **Taxon** | | 6 | 45770 | 7628.3 | 2.4085 | 0.04419 * |  |
| --- | --- | --- | --- | --- | --- | --- | --- | --- |
|  | **Interaction** | | 6 | 4447 | 741.2 | 0.234 | 0.96289 |  |
|  | **Residuals** | | 40 | 126686 | 3167.2 |  |  |  |
| **Farnesol** | | **Pollen Presence** | | 1 | 22469 | 22468.7 | 7.1819 | 0.01064 * |
|  | | **Taxon** | 6 | 38828 | 6471.3 | 2.0685 | 0.07872 . |  |
|  | | **Interaction** | 6 | 54816 | 9135.9 | 2.9202 | 0.01858 * |  |
|  | | **Residuals** | 40 | 125141 | 3128.5 |  |  |  |

**Supplementary Information S7** Component weightings of PC1 and PC2. The total variation explained by principal components one and two was 57.7% (30.9% and 26.8% respectively)

| **Floral Volatile** | **Component 1** | **Component 2** |
| --- | --- | --- |
| Hexenyl Acetate | 0.403 | 0.708 |
| Linalool | 0.683 | -0.494 |
| Caryophyllene | 0.210 | 0.762 |
| Farnesal | 0.502 | -0.074 |
| Farnesol | 0.787 | -0.090 |


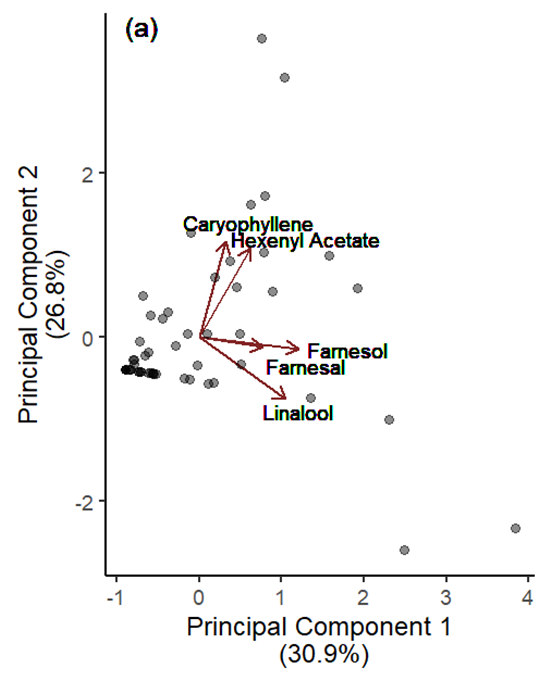

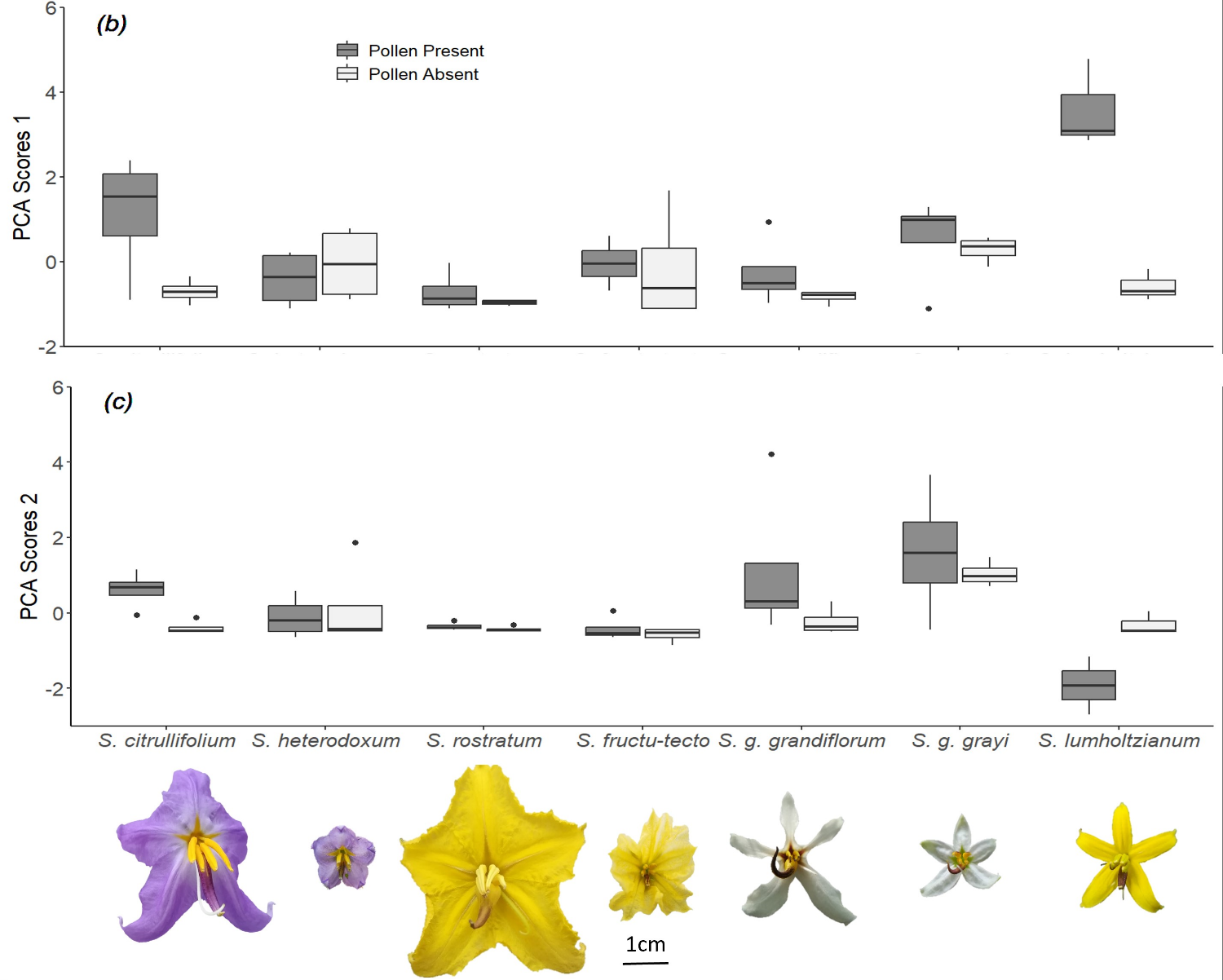
**Supplementary Information S8** Principal Component Analysis of VOC emission of *Solanum* section *Androceras*. (**a**) Biplot of PC1 and PC2 demonstrating the loadings of each of the six VOC, % variance explained by each PC presented in axis titles. (**b**) Principal Component 1, (**c**) Principal Component 2
